# Risk factors for acute neurological complications in neonatal bacterial meningitis: a retrospective cohort study

**DOI:** 10.1016/j.jped.2026.101533

**Published:** 2026-03-24

**Authors:** Fanhui Zhang, Ziyang Yu, Jiarong Pan, Tianming Yuan

**Affiliations:** a National Clinical Research Center for Child Health, Zhejiang University School of Medicine, Children's Hospital, Department of Neonatology, Hangzhou, Zhejiang, China; b Zhejiang University, College of Biomedical Engineering & Instrument Science, Key Laboratory for Biomedical Engineering of Ministry of Education, Department of Biomedical Engineering, Hangzhou, Zhejiang, China

**Keywords:** Neonate, Bacterial meningitis, Neurological complications, Risk factors, *Group B streptococcus*

## Abstract

**Objective:**

This study aimed to investigate the risk factors for acute neurological complications (ANCs) in neonates with bacterial meningitis (NBM).

**Methods:**

This retrospective study analyzed 68 neonates (aged 0–28 days) with bacterial meningitis admitted to a tertiary pediatric medical center (Children's Hospital, Zhejiang University School of Medicine, China) between November 1, 2020, and June 30, 2025. ANCs were defined as a spectrum of conditions resulting from NBM, including subdural effusion/empyema, ventriculitis, hydrocephalus, brain abscess, and encephalomalacia. The cohort comprised 48 neonates without ANCs and 20 who developed ANCs.

**Results:**

Compared with neonates without complications, those who developed ANCs were significantly older at symptom onset and demonstrated higher rates of seizures, elevated C-reactive protein (CRP) levels, increased cerebrospinal fluid (CSF) culture positivity, higher rates of *Group B Streptococcus* (GBS) infection, and more frequent dexamethasone administration. Multivariate logistic regression analysis identified seizures (OR = 10.951, 95% CI: 1.981–60.532, *p* = 0.006) and GBS infection (OR = 4.803, 95% CI: 1.072–21.523, *p* = 0.040) as independent risk factors for ANCs. Dexamethasone use (OR = 0.946, 95% CI: 0.185–4.834, *p* = 0.947) was not significant after adjustment. A predictive model incorporating both factors demonstrated good discriminatory capacity (AUC = 0.814, 95% CI: 0.686–0.942, *p* < 0.001).

**Conclusion:**

Seizures and GBS infection are independent risk factors for ANCs in NBM. Dexamethasone administration did not reduce the incidence of ANCs in this cohort.

## Introduction

Neonatal bacterial meningitis (NBM), defined as bacterial inflammation of the meninges and subarachnoid space occurring within 28 days of birth, remains a significant cause of neonatal mortality and long-term neurological impairment [1]. This condition accounts for approximately 190,000 deaths annually worldwide [2]. Among survivors, 25–50 % develop persistent neurological sequelae [3], including cerebral palsy (9 %), epilepsy (5 %), sensorineural hearing loss (3 %), and learning disabilities (8 %) by 5 years of age [4].

Acute neurological complications (ANCs) of NBM encompass a spectrum of conditions including subdural effusion/empyema, ventriculitis, hydrocephalus, brain abscess, cerebral infarction, encephalitis, cerebral venous thrombosis, and arterial stroke [5]. Early identification of neonates at high risk for ANCs is critical for implementing targeted interventions and potentially mitigating permanent neurological deficits. Nevertheless, studies investigating risk factors for ANCs in neonates with bacterial meningitis remain scarce.

Several studies have explored predictors of specific complications or mortality in infant meningitis. A retrospective study in China identified multidrug-resistant infection and C-reactive protein levels > 50 mg/L as risk factors for brain abscess in neonatal meningitis [6]. A prospective study in Angola found that elevated heart rate and the presence of seizures were independent predictors of mortality in infants under 90 days of age with bacterial meningitis [7]. Similarly, a retrospective study in Taiwan reported that seizures at onset, early-onset sepsis, and neurological complications requiring surgical intervention were independent predictors of moderate-to-severe disability or death in infants under 90 days with Gram-negative bacterial meningitis [8]. While these studies provide valuable insights, they have largely focused on either specific complications (e.g., brain abscess) or long-term/mortality outcomes, rather than comprehensively examining risk factors for the full spectrum of ANCs in the neonatal population.

Therefore, this study aimed to identify risk factors for the development of ANCs in neonatal bacterial meningitis.

## Methods

### Study design and population

This retrospective cohort study was approved by the Ethics Committee of a tertiary pediatric medical center (Children's Hospital, Zhejiang University School of Medicine, China; Approval Number: 2025-IRB-0225-*p* - 01). The requirement for informed consent was waived due to the retrospective nature of the study. The authors systematically reviewed and analyzed the medical records of all neonates (aged 0–28 days) diagnosed with bacterial meningitis who were admitted to the study center between November 1, 2020, and June 30, 2025.

### Diagnostic criteria for neonatal bacterial meningitis

According to World Health Organization (WHO) standards [9], the diagnosis of NBM was established when at least one of the following criteria was met [[10], [11], [12]]: (1) Identification of bacteria from CSF by culture or molecular testing. (2) Identification of bacteria from positive blood culture combined with CSF findings meeting at least two of the following criteria: CSF white blood cell count > 20 × 10⁶/L, CSF glucose < 1.7 mmol/L (term infants) or < 1.1 mmol/L (preterm infants), CSF protein > 1000 mg/L (term infants) or > 1500 mg/L (preterm infants). (3) Negative bacterial detection from CSF and blood with presence of clinical signs consistent with bacterial meningitis plus CSF findings meeting at least two of the following criteria: CSF white blood cell count > 100 × 10⁶/L, CSF glucose < 1.7 mmol/L (term infants) or < 1.1 mmol/L (preterm infants), CSF protein > 1000 mg/L (term infants) or > 1500 mg/L (preterm infants).

Exclusion criteria included: intraventricular hemorrhage; central nervous system infections caused by non-bacterial pathogens (herpes simplex virus, cytomegalovirus, Toxoplasma, syphilis, enterovirus, fungi); and congenital nervous system malformations.

### Assessment of acute neurological complications

ANCs were defined based on clinical and neuroimaging criteria (cranial ultrasound and/or MRI). **Ventriculitis:** Thickened, irregular, hyperechoic ependyma with striped hyperechoic areas attached to the ependyma within the ventricles, with or without ventricular dilation on ultrasound; ventricular wall enhancement on MRI. **Hydrocephalus:** Bulging or tense fontanelle, widened sutures, and ventricular dilation on ultrasound or MRI, potentially accompanied by periventricular edema. **Brain abscess:** Rim-enhancing hypointense lesion on MRI. **Subdural effusion/empyema:** Crescentic or fusiform hypoechoic collection on ultrasound; crescentic signal abnormality with dural enhancement on MRI. **Encephalomalacia:** Cystic signal changes on ultrasound or MRI.

All neuroimaging scans were independently reviewed by two experienced pediatric neuroradiologists who were blinded to clinical outcomes. Discrepancies were resolved through consensus. Neonates exhibiting one or more complications were classified as having ANCs.

### Statistical analysis

All statistical analyses were performed using SPSS version 29.0 (IBM Corporation, Armonk, NY, USA). A two-sided p-value < 0.05 was considered statistically significant. The normality of continuous variables was assessed using the Shapiro-Wilk test. Normally distributed continuous variables were presented as mean ± standard deviation (SD) and compared between groups using independent samples *t*-tests. Non-normally distributed continuous variables were expressed as median with interquartile range (IQR) and compared using the Mann-Whitney U test. Categorical variables were expressed as frequencies and percentages and compared using the chi-square test or Fisher's exact test, as appropriate.

Variables identified as clinically significant in group comparisons underwent univariate logistic regression analysis with ANC presence as the dependent variable. Variables achieving statistical significance (*p* < 0.05) in univariate analysis were entered into multivariate logistic regression using the backward stepwise method to identify independent predictors of ANCs. Potential interactions between predictors and with sex or age at onset were also analyzed. Collinearity among variables was assessed prior to multivariate modeling.

A predictive model incorporating the identified independent predictors was developed. Receiver operating characteristic (ROC) analysis was employed to evaluate the diagnostic performance of individual predictors and the combined model, with calculation of the area under the curve (AUC). Internal validation was performed using the Bootstrap method with 1000 iterations. Model calibration was assessed using the Hosmer-Lemeshow test.

## Results

### Participant demographic characteristics

A total of 68 neonates with bacterial meningitis were included for the final analysis. The overall prevalence of ANCs was 29.41 % (20/68). The incidence of specific ANCs was as follows: encephalomalacia in 17.65 % (12/68), subdural effusion/empyema in 11.76 % (8/68), hydrocephalus in 10.29 % (7/68), brain abscess in 5.88 % (4/68), and ventriculitis in 4.41 % (3/68). Notably, 14.71 % (10/68) of neonates presented with two or more concurrent complications.

Neonates who developed ANCs were significantly older at symptom onset compared to those without complications (median 14.50 days [IQR 8.25–19.00] vs. 7.50 days [IQR 2.25–14.75]; *p* = 0.027) (Table 1). No other significant demographic differences were observed between the two groups (Table 1).

**Table 1:** Comparison of demographic characteristics between neonates with bacterial meningitis with and without acute neurological complications.

	With Complications (*n* = 20)	Without Complications (*n* = 48)	t/z/χ²	p-value
Birth weight (g)	2860 (2295–3208)	2955 (2148–3335)	−0.074	0.941
Gestational age (weeks)	37.79 (35.64–39.32)	38.29 (34.47–39.61)	−0.215	0.829
Sex (female/male)	12/8	20/28	1.905	0.168
Age at onset (days)	14.50 (8.25–19.00)	7.50 (2.25–14.75)	2.205	**0.027**
Very low birth weight infants, n ( %)	0 (0.0)	3 (6.3)	1.308	0.550
Premature infants, n ( %)	6 (30.0)	16 (33.3)	0.072	0.789
Age at onset ≤3 days, n ( %)	2 (10.0)	13 (27.1)	2.396	0.199
Twins, n ( %)	2 (10.0)	4 (8.3)	0.049	1.000
Cesarean delivery, n ( %)	9 (45.0)	20 (41.7)	0.064	0.800
Premature rupture of membranes > 18 h, n ( %)	1 (5.0)	3 (6.3)	0.040	1.000
Meconium-stained amniotic fluid > Grade II, n ( %)	0 (0.0)	2 (4.2)	0.859	1.000
Intrauterine distress, n ( %)	0 (0.0)	3 (6.3)	1.308	0.550
Postnatal asphyxia, n ( %)	1 (5.0)	1 (2.1)	0.421	0.505

Note: Continuous variables with normal distribution are presented as mean ± standard deviation; continuous variables with non-normal distribution are presented as median (interquartile range). Bold values indicate statistical significance at *p* < 0.05.

### Clinical manifestations, laboratory parameters, comorbidities, and treatment

Compared with the no-complication group, neonates in the ANC group demonstrated a significantly higher incidence of seizures, defined specifically as clinically evident events (Table 2) (χ² = 20.521, *p* < 0.001), reflecting a marked difference in neurological morbidity between groups. This difference remained statistically significant after adjusting for age at onset. C-reactive protein (CRP) levels were significantly elevated in the ANC group, both before and after age adjustment (*t* = −1.978, *p* = 0.047), although the modest effect size suggests that CRP should be interpreted alongside other clinical markers. The cerebrospinal fluid (CSF) culture positivity rate was significantly higher in the ANC group, a difference that persisted after adjustment (χ² = 5.941, *p* = 0.047), indicating a greater pathogen burden in complicated cases. Similarly, the prevalence of *Group B Streptococcus* (GBS) infection was significantly higher in the ANC group, and this difference remained significant after adjustment (χ² = 15.451, *p* < 0.001), implicating GBS as a key contributor to severe neonatal meningitis. The duration of antibiotic treatment was significantly longer in the ANC group, a difference that also remained significant after adjustment (*t* = 4.075, *p* < 0.001), reflecting the increased clinical complexity and prolonged recovery period associated with complications. While the ANC group had a higher rate of dexamethasone administration (χ² = 5.785, *p* = 0.016), this difference was not significant after adjustment for age at onset (*p* = 0.054), suggesting that age at onset confounded this univariate association.

**Table 2:** Comparison of clinical manifestations, laboratory parameters, comorbidities, and treatment between neonates with bacterial meningitis with and without acute neurological complications.

Variables	With Complications (*n* = 20)	Without Complications (*n* = 48)	t/z/χ²	P₁	P₂
Clinical Symptoms					
Seizures, n ( %)	11 (55.0)	3 (6.3)	20.521	**<0.001**	**<0.001**
Apnea, n ( %)	2 (10.0)	2 (4.2)	0.868	0.575	0.503
Irritability, n ( %)	2 (10.0)	7 (14.6)	0.259	1.000	0.569
Lethargy, n ( %)	2 (10.0)	1 (2.1)	2.098	0.205	0.249
Poor feeding, n ( %)	5 (25.0)	11 (22.9)	0.034	1.000	0.730
Tachypnea, n ( %)	3 (15.0)	10 (20.8)	0.311	0.425	0.747
Fever > 72 h, n ( %)	7 (35.0)	7 (14.6)	3.599	0.097	0.118
Blood Tests					
White blood cells (× 10⁹/L)	6.88 (2.62–14.18)	11.63 (5.45–17.45)	−1.613	0.107	0.333
Platelets (× 10⁹/L)	284.50 (208.75–331.50)	169.00 (87.50–395.50)	1.623	0.105	0.725
C-reactive protein (mg/L)	87.9 ± 62.6	62.3 ± 41.6	−1.978	0.052	**0.047**
WBC 〈 5 or 〉 20 × 10⁹/L, n ( %)	8 (47.1)	15 (31.9)	1.244	0.265	0.425
Platelets < 150 × 10⁹/L, n ( %)	3 (21.4)	18 (43.9)	2.233	0.135	0.318
CRP > 50 mg/L, n ( %)	13 (65.0)	28 (58.3)	0.262	0.609	0.594
Lactate (mmol/L)	3.00 (2.40–5.60)	2.60 (2.05–3.65)	0.825	0.416	0.325
Cerebrospinal Fluid					
White blood cells (× 10⁶/L)	1163 (490–4292)	1110 (140–4144)	0.861	0.389	0.955
Glucose (mmol/L)	1.72±0.91	2.05±1.25	0.751	0.457	0.576
Protein (mg/L)	2507 (1601–3013)	2040 (1492–2605)	0.992	0.327	0.402
Microbiology					
Positive for bacteria, n ( %)	20 (100.0)	41 (85.4)	3.251	0.096	0.999
Positive CSF culture, n ( %)	16 (80.0)	23 (47.9)	5.941	**0.015**	**0.047**
*Group B Streptococcus*, n ( %)	13 (65.0)	8 (16.7)	15.451	**<0.001**	**<0.001**
*Escherichia coli*, n ( %)	3 (15.0)	12 (25.0)	0.821	0.525	0.468
*Staphylococcus*, n ( %)	1 (5.0)	5 (10.4)	0.515	0.662	0.659
Other bacteria, n ( %)	3 (15.0)	16 (33.3)	2.357	0.125	0.146
Comorbidities					
HsPDA, n ( %)	0 (0.0)	1 (2.1)	0.423	1.000	1.000
Pneumonia, n ( %)	3 (15.0)	3 (6.3)	1.344	0.349	0.292
Respiratory failure, n ( %)	2 (10.0)	6 (12.5)	0.085	1.000	0.655
NEC, n ( %)	0 (0.0)	1 (2.1)	0.423	1.000	1.000
Treatment					
Mechanical ventilation, n ( %)	2 (10.0)	7 (14.6)	0.258	1.000	0.612
TPN, n ( %)	6 (30.0)	14 (29.2)	0.005	0.945	0.733
Time from onset to appropriate antibiotics (days)	1.0 (1.0–1.0)	1.0 (1.0–1.0)	−0.903	0.367	0.214
Duration of antibiotic therapy (days)	56.00 (48.50–74.50)	28.50 (23.00–40.75)	4.075	**<0.001**	**<0.001**
Dexamethasone use, n ( %)	10 (50.0)	10 (20.8)	5.785	**0.016**	0.054

Note: Continuous variables with normal distribution are presented as mean ± deviation; continuous variables with non-normal distribution are presented as median (interquartile range). P₁: Comparison between groups with and without neurological complications; P₂: Comparison between groups with and without neurological complications after adjusting for age at onset. Bold values indicate *p* < 0.05.

WBC, White blood cell count; CRP, C-reactive protein; CSF, Cerebrospinal fluid; HsPDA, Hemodynamically significant patent ductus arteriosus; NEC, Necrotizing enterocolitis; TPN, Total parenteral nutrition.

### Risk factors for ANCs

Univariate analysis identified several potential risk factors: age at onset, seizures, CRP levels, CSF culture positivity, GBS infection, and dexamethasone use. Multivariate logistic regression analysis confirmed seizures (OR = 10.951, 95 % CI: 1.981–60.532, *p* = 0.006) and GBS infection (OR = 4.803, 95 % CI: 1.072–21.523, *p* = 0.040) as independent predictors of ANCs (Table 3). However, due to the limited sample size, these estimates exhibited wide confidence intervals and should therefore be interpreted with caution. Dexamethasone use (OR = 0.946, 95 % CI: 0.185–4.834, *p* = 0.947) was not identified as an independent predictor (Table 3). No significant interactions were found between these independent factors or between the independent factors and gender or age at onset (Table S1). An assessment of collinearity prior to multivariate modeling revealed no significant multicollinearity among the variables (Table S2).

**Table 3:** Risk Factor Analysis for Acute Neurological Complications in Neonatal Bacterial Meningitis.

Variables	Univariate Logistic Regression			Multivariate Logistic Regression		
	OR	95 % CI	P-value	OR	95 % CI	P-value
Age at onset (days)	1.076	1.003–1.154	**0.042**	1.051	0.955–1.157	0.305
Seizures, n ( %)	18.333	4.243–79.221	**<0.001**	10.951	1.981–60.532	**0.006**
C-reactive protein (mg/L)	1.010	1.000–1.022	0.060	1.004	0.990–1.019	0.569
Positive CSF culture, n ( %)	4.348	1.267–14.925	**0.020**	1.696	0.330–8.715	0.528
*Group B Streptococcus*, n ( %)	9.286	2.820–30.579	**<0.001**	4.803	1.072–21.523	**0.040**
Dexamethasone use, n ( %)	3.800	1.240–11.642	**0.019**	0.946	0.185–4.834	0.947

Note: Bold values indicate *p* < 0.05.

OR, Odds ratio; CI, Confidence interval; CS, Cerebrospinal fluid.

### Prediction model performance

**Figure 1:**
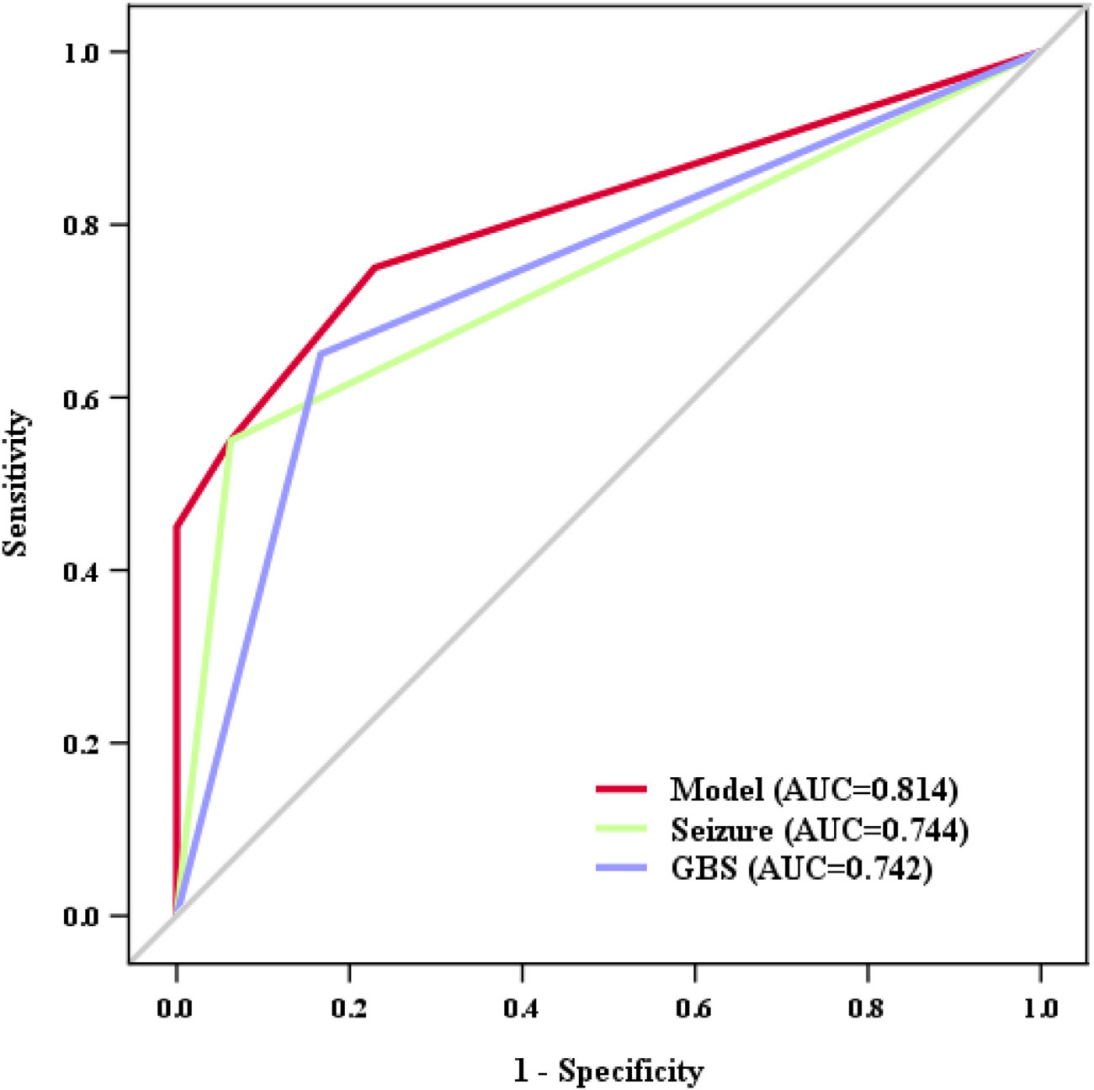
Receiver Operating Characteristic (ROC) Curve Analysis for Predicting Acute Neurological Complications in Neonatal Bacterial Meningitis. The figure displays ROC curves comparing the predictive performance of a combined model versus individual risk factors. The combined model (incorporating seizures and GBS infection) achieved an AUC of 0.814, outperforming individual predictors: seizures alone (AUC = 0.744) and GBS infection alone (AUC = 0.742).

AUC, Area Under the Curve; ROC, Receiver Operating Characteristic; GBS, *Group B Streptococcus*.

Based on the two independent predictors — seizures and GBS infection — a prediction model was constructed to identify neonates with bacterial meningitis at risk of developing acute neurological complications (Figure 1). The area under the receiver operating characteristic (ROC) curve (AUC) values for the individual predictors were: seizures (AUC = 0.744, 95 % CI: 0.599–0.889, *p* = 0.001) and GBS infection (AUC = 0.742, 95 % CI: 0.603–0.881, *p* = 0.001). The combined prediction model incorporating both seizures and GBS infection demonstrated superior predictive performance (AUC = 0.814, 95 % CI: 0.686–0.942, *p* < 0.001), with a sensitivity of 0.550 and specificity of 0.938 (Figure 1). Bootstrap validation indicated good model fit, and the Hosmer-Lemeshow test confirmed good calibration (χ² = 8.452, *p* = 0.334).

## Discussion

This study demonstrates a high incidence of ANCs (29.41 %) in neonatal bacterial meningitis and identifies seizures and GBS infection as independent risk factors for their development. A predictive model combining these factors effectively identified NBM patients at risk for acute neurological complications with good discriminatory capacity (AUC = 0.814). Dexamethasone administration did not reduce the incidence of ANCs in our cohort. These findings provide a foundation for early risk stratification and underscore the need for heightened clinical vigilance in neonates presenting with seizures or GBS infection.

The incidence of specific acute neurological complications observed in our cohort—subdural effusion/empyema (11.76 %), ventriculitis (4.41 %), hydrocephalus (10.29 %), brain abscess (5.88 %), and encephalomalacia (17.65 %) — generally aligns with ranges reported in previous literature. The subdural effusion incidence approximates the 11 % reported for neonatal bacterial meningitis and the 7–13 % range for Gram-negative meningitis [13], [14]. While ventriculitis has been reported in 5–10 % of neonatal meningitis cases [15], hydrocephalus in approximately 25 % [13,15], and brain abscess in 10 % of neonatal meningitis patients[16] or 10–20 % in Gram-negative meningitis [15], [16], our observed rates for these specific complications were somewhat lower. These variations may reflect differences in inclusion criteria, age distribution, pathogen profiles, or treatment protocols across studies.

Our finding of a significantly higher incidence of seizures in the ANC group is consistent with extensive prior research linking seizures to adverse outcomes in NBM. Multiple studies have identified seizures as a predictor of mortality or neurodevelopmental disability in neonatal bacterial meningitis[17,18]. Specifically, seizures have been demonstrated to be an independent risk factor for adverse outcomes in neonates with Gram-negative bacterial meningitis [8], and studies across diverse populations confirm their independent association with poor outcomes in bacterial meningitis overall [19]. While seizures were identified as an independent predictor of ANCs in our cohort, it is important to consider the nature of this association. This relationship may reflect, at least in part, seizures serving as an early clinical manifestation of evolving neurological injury rather than as a truly independent causal factor. Given the observational design of this study, definitive conclusions regarding causality are precluded. Accordingly, seizures should be interpreted primarily as an early indicator of disease severity that may facilitate risk stratification, rather than as a modifiable target for intervention.

This study provides evidence that GBS infection is significantly associated with an increased risk of acute neurological complications in NBM. This finding aligns with the well-documented severe neurological consequences of GBS meningitis. Prospective studies following neonatal GBS meningitis survivors have documented high rates of neurological dysfunction (49 % at 5 years) [20], neurodevelopmental impairment (25 % moderate/mild, 19 % severe) [21], and disabilities persisting beyond 18 months (32 %, with nearly one-fifth being moderate or severe) [22]. Our results suggest that this propensity for neurological damage manifests acutely as a higher risk of the complications studied.

The authors found no evidence supporting the use of dexamethasone for ANC prevention in NBM. The role of adjunctive dexamethasone in neonates remains controversial and inadequately studied compared to older populations. While randomized controlled trials and meta-analyses demonstrate benefits (reduced hearing loss and neurological sequelae) in pediatric and adult bacterial meningitis [[23], [24], [25]], and cohort studies suggest improved outcomes in certain settings [[26], [27], [28], [29]], concerns exist regarding potential adverse effects, such as reduced survival in neuro-listeriosis [30]. Neonatal randomized controlled trials remain limited and inconclusive: one found no difference in outcomes, including complications [31], while another reported reduced mortality and inflammation [32]. A meta-analysis indicated reduced hearing loss but no benefit for mortality or severe neurological outcomes [33]. Our data contribute specifically to the ANC context, indicating no protective effect of dexamethasone administration.

ANCs profoundly impact the long-term prognosis of NBM. The absence of simple predictive models in the literature underscores the utility of our tool, which integrates readily assessable clinical (seizures) and microbiological (GBS) features. Early identification of high-risk neonates could prompt intensified antibiotic therapy, vigilant neuroimaging surveillance, and aggressive complication management, potentially attenuating long-term neurological deficits. Nevertheless, while the model exhibits reasonable discriminatory ability, its relatively low sensitivity (55.0 %) indicates that it would fail to identify nearly half of the neonates who ultimately develop ANCs, limiting its utility as a standalone screening tool in clinical practice. The modest sample size and limited number of predictors available for model development likely contributed to this limitation and may have introduced optimism into the performance estimates. Given its high specificity (93.8 %), the model may be best employed as an adjunct to clinical judgment — helping to identify a subset of patients at particularly high risk — while risk assessment in screen-negative individuals should continue to rely on comprehensive clinical evaluation. Accordingly, external validation in larger, independent cohorts is essential before this model can be considered for clinical application.

This study has several limitations. First, its retrospective design introduces the potential for selection bias. Additionally, reliable data on antibiotic administration prior to lumbar puncture were unavailable, precluding assessment of this potential confounder, which may influence CSF culture positivity rates. Second, the modest sample size limits statistical power for subgroup analyses and detection of smaller effect sizes, and increases the risk of overfitting in our multivariate model. Third, external validation of the predictive model in independent cohorts is pending. Future prospective multicenter studies with larger cohorts are needed to validate and refine this model and explore additional risk factors.

## Conclusion

This study identified an incidence of acute neurological complications in neonatal bacterial meningitis of 29.41 %. Seizures and GBS infection were identified as independent risk factors for these complications. Dexamethasone administration did not reduce the incidence of ANCs in this cohort. These findings may help clinicians identify high-risk neonates who require closer monitoring and more aggressive management strategies. However, the results should be interpreted with caution given the study's limitations, including its retrospective design, modest sample size, and single-center setting. The predominant role of GBS observed in this cohort may reflect regional pathogen prevalence and local clinical practices, potentially limiting generalizability to populations with different epidemiological profiles.

## Ethics approval and consent to participate

This study was conducted in accordance with the Declaration of Helsinki concerning Ethical Principles for Medical Research Involving Human Subjects. To protect participant privacy, names were not used and data confidentiality was maintained throughout the study. Ethical approval was obtained from the Institutional Review Board of Children's Hospital, Zhejiang University School of Medicine (Approval Number: 2025-IRB-0225-*p* - 01). The requirement for informed consent was waived due to the retrospective nature of the study.

## Funding

This work was supported by the 10.13039/501100017531Zhejiang Province Health Project (Grant No 2021KY764). The funding organization had no role in the study design, data collection and analysis, decision to publish, or preparation of the manuscript.

## Authors' contributions

FZ: Conceptualization, Data curation, Formal analysis, Investigation, Methodology, Project administration, Visualization, Writing - original draft, Writing - review & editing; ZY: Data curation, Formal analysis, Investigation, Methodology, Project administration, Validation, Writing - review; JP: Data curation, Formal analysis, Methodology, Resources, Supervision, Validation, Visualization, Writing - review; TY: Conceptualization, Methodology, Resources, Supervision, Validation, Writing - review.

## Data availability statement

Data are available on reasonable request (email: yuantianming @zju.edu.cn).

## Conflicts of interest

The authors declare no conflicts of interest.

## Appendix Supplementary materials

Image, application 1

## Footnotes

Supplementary material associated with this article can be found in the online version at doi:10.1016/j.jped.2026.101533.
