# Supplementary material for: Risk factors for acute neurological complications in neonatal bacterial meningitis: a retrospective cohort study
Source: J Pediatr (Rio J). 2026 Mar 24;102(3):101533. doi: 10.1016/j.jped.2026.101533 (PMC13049943; doi:10.1016/j.jped.2026.101533)
Supplement: Supplementary file 1 [file mmc1.docx]

**JPED-D-25-00496_Supplementary Material**

| **Table S1** Analysis of interactions between variables. |
| --- |

| Variables | *P-*interaction |
| --- | --- |
| Age at onset * Seizures | 0.656 |
| Age at onset * *Group B Streptococcus* | 0.185 |
| Seizures * *Group B Streptococcus* | 0.185 |
| Age at onset * Sex | 0.108 |
| Seizures * Sex | 0.531 |
| *Group B Streptococcus* * Sex | 0.140 |

**Table S2** Collinearity analysis between variables.

| Variables | Tolerance | Variance inflation factor |
| --- | --- | --- |
| Age at onset (days) | 0.875 | 1.143 |
| Seizures, n (%) | 0.752 | 1.330 |
| C-reactive protein (mg/L) | 0.891 | 1.122 |
| Positive CSF culture, n (%) | 0.777 | 1.287 |
| *Group B Streptococcus*, n (%) | 0.749 | 1.335 |
| Dexamethasone use, n (%) | 0.760 | 1.315 |
